# Ontogenetic Trophic Differentiation in the Amur Softshell Turtle (*Pelodiscus maackii*) From a Temperate Large‐River System

**DOI:** 10.1002/ece3.74221

**Published:** 2026-08-26

**Authors:** Xiaochen Hou

**Affiliations:** ^1^ School of Medicine and Health Tonghua Normal University Tonghua China

**Keywords:** Bray–Curtis, niche overlap, ontogenetic diet shift, *Pelodiscus maackii*, PERMANOVA, piscivory, size‐structured food web, stomach content analysis

## Abstract

The Amur softshell turtle ( *Pelodiscus maackii* ) has undergone severe population declines across northeastern China, yet its trophic ecology remains poorly understood. Because ontogenetic dietary shifts can generate size‐structured functional roles within food webs, clarifying diet across life stages is critical for both ecological inference and conservation. We examined diet composition and ontogenetic trophic differentiation in a remnant population of *P. maackii* from the midstream Ussuri River (China side) using stomach content analysis. Samples were obtained opportunistically from incidental fishery bycatch and market‐derived remains collected between July 2022 and October 2025. Diet was quantified using frequency of occurrence and volumetric contribution of five functional prey categories and analyzed with multivariate ordination and permutation‐based tests, and niche overlap indices. Across 41 individuals with nonempty stomachs, diet composition differed strongly among ontogenetic stages, whereas no sex‐specific differences were detected after accounting for body mass. Juveniles exhibited diverse, invertebrate‐dominated diets, particularly freshwater snails, while fish increased sharply with body size and dominated the diets of subadult and adult turtles. Dietary richness and within‐group variability declined with ontogeny, and niche overlap was lowest between juveniles and adults but high among older stages. Together, these results demonstrate pronounced size‐structured trophic differentiation in *P. maackii* , indicating that population declines or size‐selective losses may have disproportionate consequences for riverine food‐web structure and function.

## Graphical abstract

We investigated ontogenetic variation in diet composition of the Amur softshell turtle ( *Pelodiscus maackii* ) from a temperate large‐river system using stomach content analyses of field‐collected individuals. Dietary composition differed markedly across body‐size classes, indicating pronounced ontogenetic trophic differentiation and shifts in resource use with growth. These patterns highlight size‐dependent ecological roles of *P. maackii* and have important implications for understanding population vulnerability and conservation management in river systems affected by fisheries.

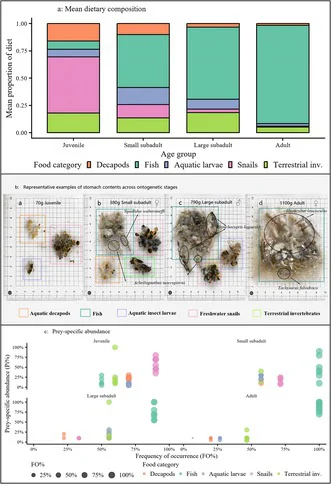

## 1 Introduction

The Amur softshell turtle ( *Pelodiscus maackii* ; hereafter ASTs) is a freshwater chelonian species historically distributed across northeastern China, the Russian Far East, the Korean Peninsula, and Japan (Brandt 1857; Stejneger 1907). Despite its early description in the scientific literature, ecological research on this species remains remarkably limited. Existing studies, primarily conducted by Russian researchers, have focused largely on population surveys and distributional records (Cherepanov 1990; Tarasov and Adnagulov 1999; Maslova 2002; Adnagulov and Maslova 2003; Yu 2018). Many of these works are outdated, difficult to access, and provide little information on the ecological requirements of ASTs in detail.

In northeastern China, ASTs populations have declined dramatically over recent decades, with most populations now considered extinct (Hou and Shi 2025). To date, only two studies by Hou and Shi (2024, 2025) have examined the population size and home range of a remnant ASTs population in the midstream Ussuri River. However, these studies are insufficient to explain the severe and widespread population decline observed across northeastern China or to assess the potential ecological consequences of ASTs extirpation from local riverine ecosystems (Lovich et al. 2018). Consequently, there is an urgent need for comprehensive ecological investigations of this species, particularly those addressing its functional role within freshwater food webs.

One of the most critical knowledge gaps concerns the dietary ecology of ASTs. Dietary studies in turtles and tortoises are fundamental for understanding their ecological roles and the mechanisms underlying population dynamics (Bjorndal and Bolten 2003). As long‐lived ectotherms exhibiting diverse trophic strategies, chelonians often function as important consumers in freshwater and terrestrial ecosystems, influencing energy flow, nutrient cycling, and community structure (Heppell and Crowder 1996). Feeding habits directly affect growth rates, reproductive output, and habitat use, making diet a central determinant of individual fitness and demographic patterns (van der Have and de Jong 1996; Moll and Moll 2004; Congdon et al. 2011). Moreover, many chelonian species are currently experiencing rapid population declines due to habitat loss, overexploitation, and environmental degradation (Hawkes et al. 2007; Sung et al. 2013; Rhodin et al. 2018), further underscoring the importance of accurate knowledge of their nutritional ecology. Robust dietary information not only clarifies species‐specific ecological niches but also provides essential guidance for conservation planning, habitat management, and restoration efforts.

Despite this importance, the diet of ASTs remains almost entirely undocumented. Studies of other softshell turtle species have revealed substantial interspecific and intraspecific variation in feeding strategies: some species are omnivorous (Jensen and Das 2008; Mahoney and Lindeman 2016; Kong et al. 2022), whereas others are primarily carnivorous (Cochran and McConville 1983; Works and Olson 2018); some exhibit sex‐specific dietary differences (Plummer and Farrar 1981; Mahoney and Lindeman 2016); and diet composition may vary markedly across geographic regions within a single species (Cochran and McConville 1983; Mahoney and Lindeman 2016; Works and Olson 2018; Kong et al. 2022). Ontogenetic variation in diet has been documented in several chelonian species, such as green turtles ( *Chelonia mydas* ) (Arthur et al. 2008; Howell et al. 2016), where juveniles and adults exploit different prey types. However, direct evidence for such shifts in softshell turtles remains limited. In long‐lived reptiles, dietary composition often shifts predictably with ontogeny, as changes in body size, morphology, and energetic demands alter prey accessibility and trophic position (Werner and Gilliam 1984; Bjorndal 1997).

In this study, we investigated the dietary composition of a remnant population of ASTs in the Ussuri River, with a particular focus on ontogenetic variation in feeding patterns. Specifically, we examined how diet composition and trophic niche structure varied among body size classes and between sexes using stomach content analysis combined with multivariate statistical approaches. By clarifying the trophic ecology of ASTs, this study aims to provide a functional ecological framework for understanding the role of this species in freshwater ecosystems and to inform conservation strategies for this rapidly declining softshell turtle species in northeastern Asia.

## 2 Materials and Methods

### 2.1 Study Area

**FIGURE 1:**
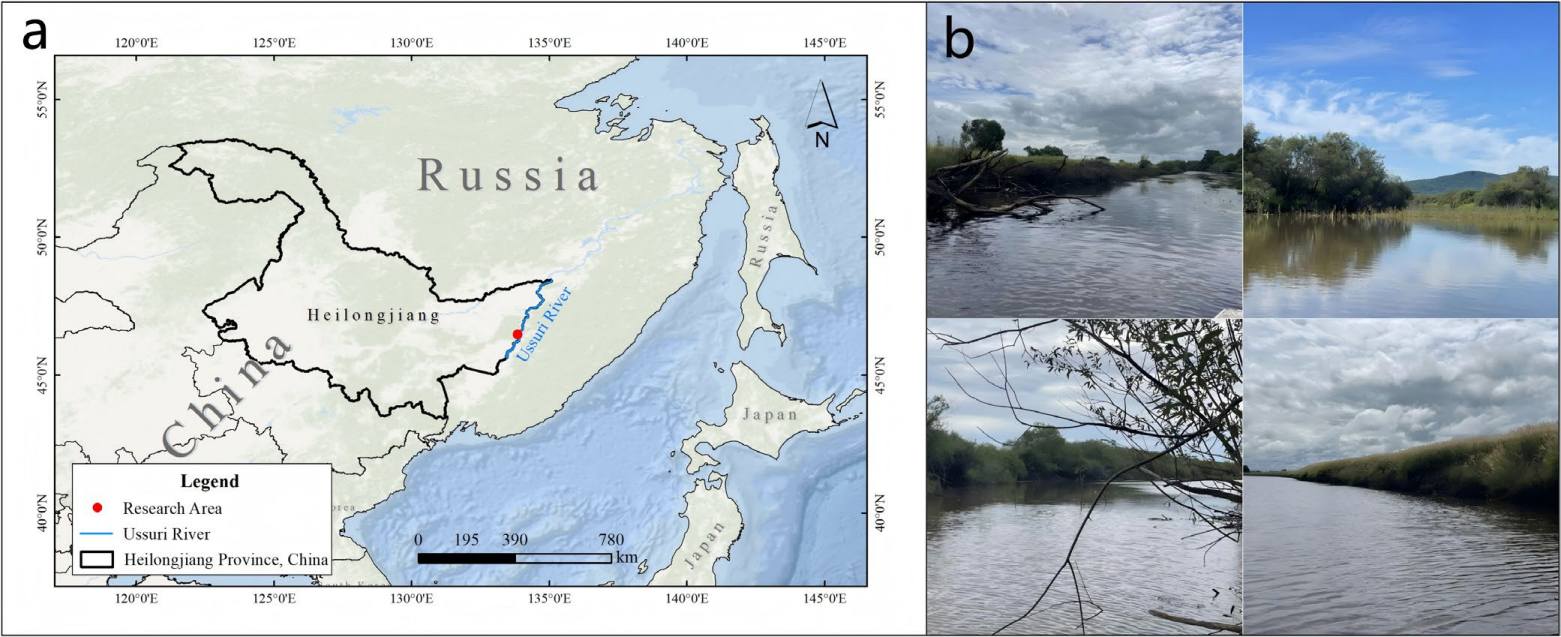
Location of the study area in Heilongjiang Province, China (a) and representative habitat along the Ussuri River (b).

This research was conducted as part of a broader ecological study in the vicinity of Jewellery Island (45°17′–45°52′ N, 133°28′–133°47′ E) within the Ussuri River, Heilongjiang Province, China (Figure 1a). The region is characterized by a temperate monsoon climate, with long, extremely cold winters during which mean temperatures remain below −20°C and may occasionally decline to −40°C. Summers are short, warm, and humid, with average temperatures of 18°C–22°C, and most annual precipitation occurs during this period. Spring and autumn are brief and marked by pronounced diurnal temperature fluctuations. The study area lies within a transitional zone between mixed forests and temperate forest‐steppe, with extensive wetlands along the main river and connected lentic channels, where dense aquatic vegetation provides habitats for diverse aquatic and benthic prey (Figure 1b).

The Ussuri River in this region is a large, complex system, with river width varying substantially within the 80–100 km sampling range upstream and downstream of Jewellery Island. The river reaches a maximum width of approximately 800 m in some sections and narrows to 150–200 m in others. Water depth also varies with season, ranging from 7–12 m depending on high‐ or low‐water periods. The river contains numerous small islands, side channels, and connected lentic habitats, creating a mosaic of flow conditions and habitats suitable for aquatic prey. The study area is sparsely populated and retains extensive tracts of relatively undisturbed forest. Several villages, fishing locations, and county towns—most notably Raohe Town and Hutou Town—are scattered along this stretch of the river, where residents primarily engage in agriculture and fishing. The Ussuri River forms the international boundary between China and Russia, with each country sharing approximately half of its waters. The opposite bank is largely uninhabited and dominated by undisturbed forest (Figure 1a).

### 2.2 Data Collection

Samples were collected as part of a broader ecological study of ASTs conducted in the Jewellery Island area of the Ussuri River. Field‐based sampling occurred between 2022 and 2023, with additional samples obtained through established local contacts after departure from the field base; the complete sampling period spanned July 2022 to October 2025.

ASTs samples were collected opportunistically as incidental bycatch from fishing activities carried out by licensed fishermen in the Ussuri River. These fishermen employ various fishing methods, including the use of different types of hoop nets in lentic channels. Although their target species were economically valuable fish rather than ASTs, these hoop nets—without ventilation openings—were the primary means by which ASTs were captured. If nets were checked infrequently, trapped turtles occasionally drowned. Fishermen typically discarded these drowned individuals, but we contacted local fishermen extensively, and when they encountered drowned ASTs, we collected the specimens from their locations. When we were not present at the field site, our local contacts assisted in recovering these specimens and shipped them to us frozen. Most of our study samples were obtained in this way. Additional samples were obtained from local markets and consisted exclusively of internal organs remaining after local consumption. No individuals were captured, killed, or handled intentionally for research purposes, and no incentives or guidance were provided to fishermen, vendors, or restaurants to influence the capture or use of ASTs. At the time of the study, no local regulations prohibited the capture or consumption of ASTs, and researchers had no legal authority to intervene in fishing or market activities (Figure S1). Samples were collected at fishing sites, settlements, and towns distributed within approximately 80–100 km upstream and downstream of the field base. Whenever intact specimens were available, standard morphometric measurements were taken following established protocols for turtles (Ernst and Barbour 1989; Iverson 1992). When only internal organs were obtained following local consumption, body mass measurements and photographs of the abdomen (including the tail) were requested for data recording and sex determination. Sex determination was based on a suite of external morphological traits described for softshell turtles (Plummer 1977a; Ernst and Lovich 2009). Secondary sexual characteristics were apparent in individuals with body mass ≥ 200 g in our samples; sex could not be reliably determined below this threshold. For each individual, body mass was recorded, and sex was determined when possible. Stomachs were removed and preserved in 95% ethanol or frozen at −20°C prior to laboratory examination. Individuals with empty stomachs were excluded from dietary analyses. Stomachs were classified as empty if no recognizable food items were present; such individuals were excluded from dietary analyses. All other stomachs, regardless of the absolute volume of food, were considered nonempty. Because turtles consume variable amounts and stomach fullness cannot be easily standardized, we did not assign arbitrary fullness scores (e.g., 0–10). Instead, volumetric proportions (%V) of each prey category were calculated for each individual, providing a robust measure of dietary composition and ontogenetic variation. Stomach contents were sorted and examined under a dissecting microscope (40–100×) and identified to the lowest possible taxonomic level based on diagnostic morphological characteristics. Identified prey items were then assigned to predefined food categories, including fish, aquatic decapods, aquatic insect larvae, freshwater snails, and terrestrial invertebrates.

All stomach contents were preserved in 95% ethanol and kept hydrated throughout laboratory processing. Volumes were measured using the water displacement method, with no samples dried, so that %V reflected the hydrated amounts of prey, minimizing biases associated with dry‐mass measurements of soft‐bodied and highly hydrated prey items (Hyslop 1980; Amundsen et al. 1996).

Seasonal and spatial comparisons were not conducted because the Ussuri River is located at a high latitude, resulting in a very short annual activity period for ASTs. Most drowned individuals were collected during the summer months (June–August), when water temperatures are highest and turtle activity and the risk of incidental drowning peak. In contrast, captures and drowning events are rare during cooler periods (April–May and September–October), when low water temperatures substantially reduce turtle activity and decrease the likelihood of trapped ASTs drowning. In addition, precise capture locations were unavailable for many market‐derived samples. Consequently, dietary analyses were conducted on pooled samples.

### 2.3 Data Analysis

Dietary analyses comprised a series of descriptive, multivariate, and regression‐based approaches designed to characterize ontogenetic variation in diet composition. Dietary composition was first quantified using three complementary metrics: frequency of occurrence (FO), proportional volumetric contribution, and an index of relative importance (IRI). For each food category and age group, FO was calculated as the percentage of individuals in which a given food item was present in the stomach. Proportional contribution was quantified as the mean volumetric proportion of each food category across individuals within an age group, together with standard errors and minimum–maximum values. An index of relative importance was calculated as IRI = FO × mean volumetric proportion (Pinkas et al. 1971). These metrics were used to summarize dietary composition within each age group and to provide a quantitative basis for subsequent analyses (Table 1).

**TABLE 1:** Ontogenetic variation in diet composition of the Amur softshell turtle ( *Pelodiscus maackii* ).

Dietary group	Juvenile					Small subadult					Large subadult					Adult				
	FO	IRI	% of biomass			FO	IRI	% of biomass			FO	IRI	% of biomass			FO	IRI	% of biomass		
			Mean ± SE	Min	Max			Mean ± SE	Min	Max			Mean ± SE	Min	Max			Mean ± SE	Min	Max
Fish	50	3.8	7.5 ± 3.3	0	30	100	48.6	48.6 ± 10.6	10	90	88.9	58.8	66.1 ± 10	0	100	100	90.1	90.1 ± 2.5	70	100
Aquatic decapods	70	11.2	16 ± 3.9	0	30	57.1	5.7	10 ± 3.8	0	20	22.2	0.7	3.3 ± 2.4	0	20	20	0.3	1.7 ± 0.9	0	10
Aquatic insect larvae	70	4.9	7 ± 2	0	20	57.1	9	15.7 ± 5.7	0	30	55.6	4.9	8.9 ± 3.5	0	30	26.7	0.6	2.3 ± 1.1	0	10
Terrestrial invertebrates	60	10.8	18 ± 9.6	0	100	57.1	7.8	13.6 ± 5.6	0	40	55.6	10.2	18.3 ± 10.6	0	100	46.7	2.4	5.2 ± 2.1	0	30

*Note:* % biomass indicates the proportional contribution of each dietary category to total stomach contents. Ontogenetic groups were defined based on body mass, juveniles (0–200 g), small subadults (200–500 g), large subadults (500–900 g), and adults (> 900 g).

Abbreviations: FO (%), frequency of occurrence; IRI, index of relative importance.

Prey items were grouped into five broad dietary categories (fish, aquatic decapods, aquatic insect larvae, freshwater snails, and terrestrial invertebrates) based on functional and nutritional similarity. Although multiple prey taxa could be distinguished within some categories, taxonomic resolution varied among prey groups and individuals, particularly for highly digested items. Given that ASTs inhabit a temperate river system with relatively limited prey diversity, this categorical framework was adopted to facilitate robust statistical analyses and to emphasize ecologically meaningful patterns in dietary composition and ontogenetic dietary shifts.

Specimen body mass ranged from 30–2800 g. Individuals were grouped into four ontogenetic stages for descriptive purposes: Juveniles (*n* = 10), Small Subadults (*n* = 7), Large Subadults (*n* = 9), and Adults (*n* = 15). Adults were defined as individuals with body mass ≥ 0.9 kg, based on previous observations (Hou and Shi 2024). The other three stages (Juveniles, Small Subadults, and Large Subadults) were arbitrarily assigned primarily based on observed patterns in dietary composition, reflecting ontogenetic changes in food use. These groupings were intended for descriptive purposes to illustrate dietary differences among stages. Multivariate patterns in dietary composition were examined using Bray–Curtis dissimilarities calculated from volumetric proportions of the five dietary categories. Principal coordinates analysis (PCoA) was used to visualize dietary similarity among individuals. Differences in dietary composition among age groups, sexes, and body size were tested using permutational multivariate analysis of variance (PERMANOVA) (Anderson 2001) implemented with the adonis2 function in the vegan package (Oksanen et al. 2022). For sex‐based comparisons, analyses were restricted to subadult and adult individuals with known sex, with body mass included as a covariate. Pairwise PERMANOVA comparisons were conducted when overall effects were significant, and *p*‐values were adjusted using the Holm correction.

Homogeneity of multivariate dispersion was assessed using the betadisper function to confirm that significant PERMANOVA results were not driven by differences in within‐group dispersion. Distances to group centroids and pairwise Bray–Curtis dissimilarities were summarized descriptively (Figure S2).

Dietary niche overlap among age groups was quantified using Pianka's niche overlap index (Pianka 1973) and the Morisita–Horn index (Horn 1966). Observed Pianka values were compared with null distributions generated by randomizing resource use within individuals (999 permutations). Morisita–Horn indices were calculated as complementary measures, with uncertainty estimated using bootstrap resampling (1000 iterations).

All statistical analyses were conducted in R version 4.5.2 (R Core Team 2024), primarily using the packages tidyverse (Wickham et al. 2019) and vegan. Statistical significance was assessed at *α* = 0.05.

## 3 Results

**FIGURE 2:**
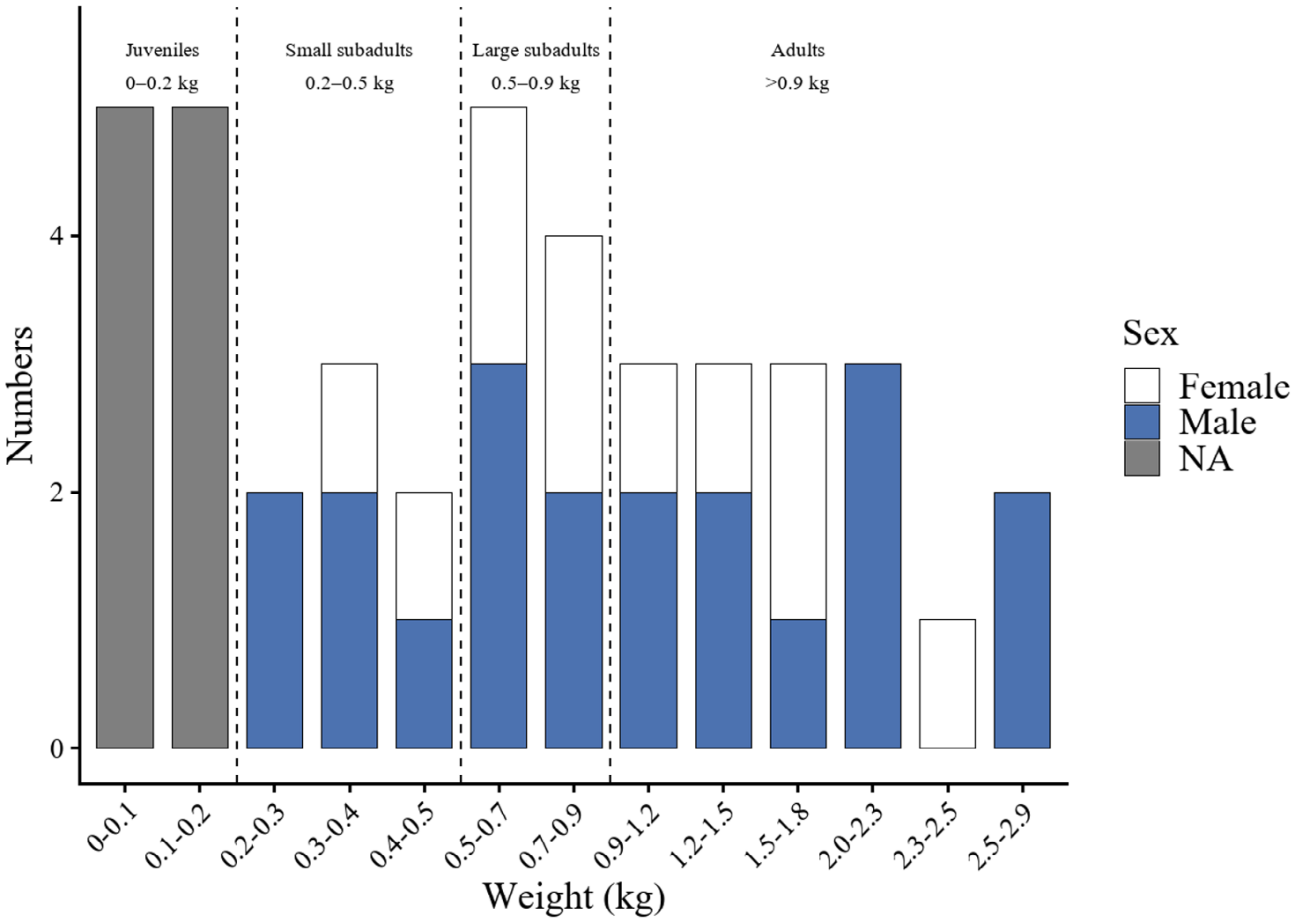
Distribution of body mass and sex composition of Amur softshell turtles ( *Pelodiscus maackii* ) included in the dietary analyses, grouped by ontogenetic stage.

Due to the low abundance of ASTs and the limited availability of suitable specimens, a total of 41 individuals with nonempty stomachs were included in the dietary analyses, collected opportunistically over 4 years. Sex could not be reliably determined for juveniles, whereas both males and females were represented among subadult and adult individuals. The distribution of individuals across body‐mass intervals and sex categories is shown in Figure 2.

Dietary composition varied substantially among ontogenetic stages (Table 1). Juveniles exhibited a diverse diet dominated by freshwater snails, which occurred in 90% of stomachs and contributed the largest proportion of dietary biomass (mean %V = 0.5 ± 0.1, IRI = 46.4). Aquatic decapods and aquatic insect larvae were also frequent components of juvenile diets (FO = 70% for both), whereas fish occurred in only half of juvenile individuals and contributed relatively little to dietary biomass (FO = 50%, mean %V = 0.1 ± 0.0).

**FIGURE 3:**
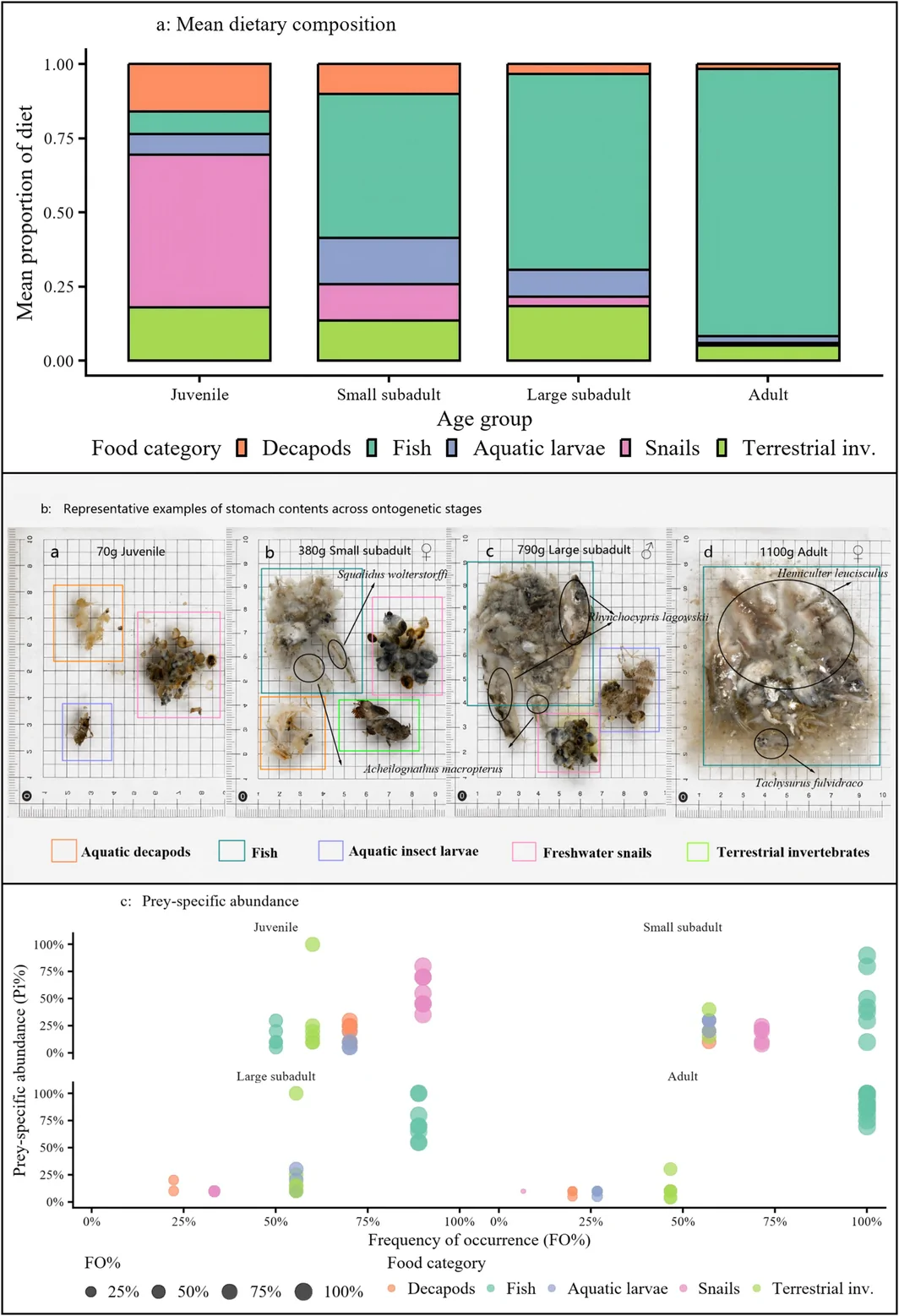
Mean dietary composition (a), representative examples of stomach contents (b), and prey‐specific abundance (c) of Amur softshell turtle across four ontogenetic stages. Panel (b) shows one representative individual from each stage, with body mass, sex, prey categories, and identifiable fish taxa indicated.

In contrast, fish became increasingly dominant with increasing body size and accounted for the majority of dietary biomass in larger individuals. Fish occurred in all small subadult and adult individuals (FO = 100%) and were also present in almost all large subadults, with the exception of one individual whose stomach contained a very large earthworm and some unidentifiable digested material. Biomass contribution of fish showed a marked increase from small subadults (mean %V = 0.5 ± 0.1, IRI = 48.6) to large subadults (mean %V = 0.6 ± 0.2, IRI = 52.3) and adults (mean %V = 0.9 ± 0.0, IRI = 90.1). Aquatic decapods contributed substantially to juvenile diets and were also common in small subadults, but their occurrence declined sharply with body size, appearing in 22% of large subadult and 20% of adult stomachs, and contributing negligibly to dietary biomass in both stages. Aquatic insect larvae, freshwater snails, and terrestrial invertebrates decreased progressively in both frequency of occurrence and biomass contribution with ontogenetic development (Table 1). Ontogenetic dietary shifts in ASTs are illustrated in Figure 3. Figure 3a shows mean dietary composition (%V), with juveniles relying mainly on freshwater snails and terrestrial invertebrates, and fish increasing in importance in large subadults and adults. Figure 3b presents stomach contents from one representative individual per ontogenetic stage, selected to reflect the typical prey composition observed within that stage. Figure 3c shows prey‐specific abundance (Pi%) versus frequency of occurrence (FO%) for individual turtles, following Amundsen et al. (1996), confirming the shift from invertebrate‐dominated diets in juveniles to fish‐dominated diets in older turtles. At finer taxonomic resolution, prey taxa spanning fishes, aquatic decapods, aquatic insect larvae, freshwater snails, and terrestrial invertebrates were identified across all age classes, with detailed prey lists and age‐specific frequencies of occurrence provided in Table S1.

**FIGURE 4:**
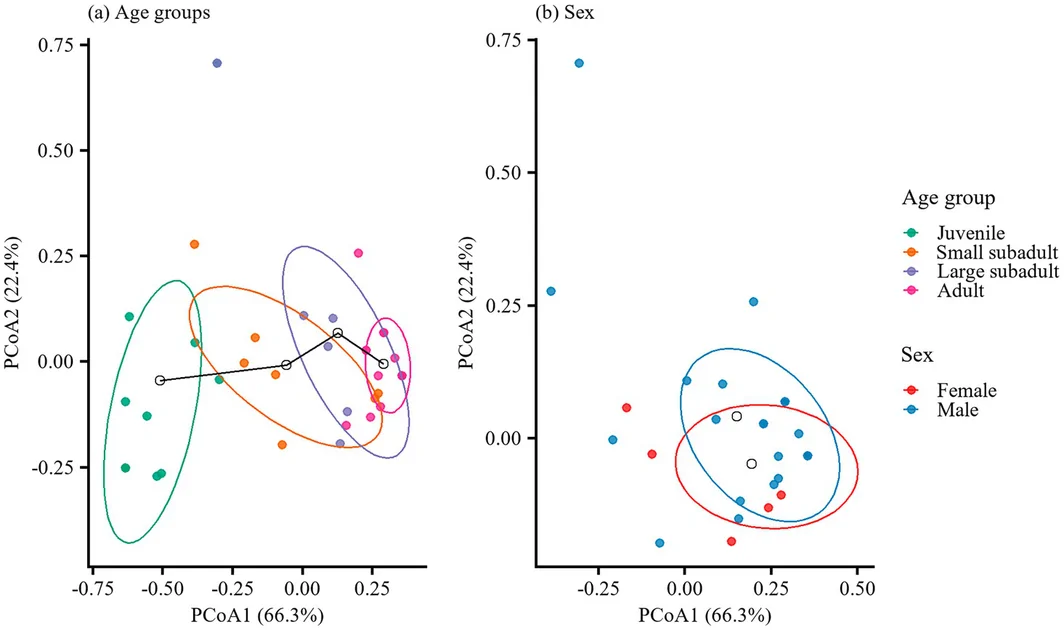
Principal coordinates analysis (PCoA) of dietary composition based on Bray–Curtis dissimilarities. (a) Ordination of individuals grouped by ontogenetic stage, with black lines connecting group centroids to illustrate the sequential change in dietary composition across successive age classes. (b) Ordination of subadult and adult individuals grouped by sex.

Multivariate analyses further supported pronounced ontogenetic structuring of dietary composition. Principal coordinates analysis (PCoA) based on Bray–Curtis dissimilarities revealed clear separation among age groups, with juveniles occupying a distinct region of ordination space and centroids of successive age groups forming an ordered trajectory along the first PCoA axis (Figure 4a). The first two PCoA axes explained 66.3% and 22.4% of the total variation in dietary composition, respectively. In contrast, males and females showed extensive overlap in ordination space (Figure 4b).

Consistent with ordination results, permutational multivariate analysis of variance identified significant differences in dietary composition among age groups (PERMANOVA, pseudo‐*F* = 5.94, *R* ^2^ = 0.30, *p* = 0.001; Table S2). Body mass explained a significant proportion of variation in dietary composition when sex was included as a covariate (pseudo‐*F* = 10.44, *R* ^2^ = 0.26, *p* = 0.001), whereas sex had no significant effect after accounting for body mass (pseudo‐*F* = 1.38, *R* ^2^ = 0.03, *p* = 0.253). Consequently, males and females were pooled in subsequent analyses. Pairwise PERMANOVA comparisons indicated significant dietary differences between juveniles and all older age classes, as well as between small subadults and adults (Table S2).

Multivariate dispersion differed significantly among age groups (beta‐dispersion permutation test, *F* = 4.14, *p* = 0.013; Figure S2a), indicating age‐related differences in within‐group variability of dietary composition. Mean Bray–Curtis distances to group centroids were highest in small subadults (0.304 ± 0.112) and juveniles (0.276 ± 0.228), intermediate in large subadults (0.261 ± 0.242), and lowest in adults (0.084 ± 0.077), suggesting a progressive reduction in dietary variability with ontogenetic development (Figure S2a). Pairwise Bray–Curtis dissimilarities increased with ontogenetic separation, with the greatest dissimilarities observed between juveniles and adults, whereas comparisons between adjacent age classes showed lower dissimilarity (Figure S2b; Table S2).

Dietary niche overlap differed markedly among ontogenetic stages (Table 2). Pianka's niche overlap was lowest between juveniles and adults (Pianka_O = 0.162), intermediate between juveniles and subadult stages (Pianka_O = 0.278–0.472), and consistently high among subadult and adult stages (Pianka_O = 0.906–0.971). Null model analyses indicated that overlap involving juveniles was significantly lower than expected under random resource use (*p*(obs > null) = 0.000), whereas overlap among older age classes showed little deviation from null expectations.

**TABLE 2:** Dietary niche overlap among ontogenetic stages of Amur softshell turtles based on Pianka's niche overlap index and the Morisita–Horn index.

Comparison	Pianka's *O*			Morisita‐Horn Index	
	*O* Value	*p* (obs < null)	*p* (obs > null)	Morisita_Horn	MH 95% CI
Juvenile vs. Small subadult	0.472	1.000	0.000	0.472	0.254–0.68
Juvenile vs. Large subadult	0.278	1.000	0.000	0.273	0.115–0.465
Juvenile vs. Adult	0.162	1.000	0.000	0.147	0.062–0.255
Small subadult vs. Large subadult	0.961	0.076	0.924	0.936	0.687–0.982
Small subadult vs. Adult	0.906	0.293	0.707	0.806	0.549–0.945
Large subadult vs. Adult	0.971	0.031	0.969	0.938	0.722–0.993

Morisita–Horn indices exhibited the same pattern, with low overlap between juveniles and adults (0.147; 95% CI = 0.062–0.255) and high overlap among subadult and adult stages (0.806–0.938; Table 2), indicating increasing dietary similarity and niche convergence with ontogenetic development.

## 4 Discussion

### 4.1 Ontogenetic Shifts in Diet and Trophic Niche of ASTs

Our study provides the first detailed characterization of dietary composition and ontogenetic trophic differentiation in the Amur softshell turtle (ASTs) from a temperate large‐river system. Across multiple complementary analytical approaches, including descriptive stomach content metrics, multivariate ordination, permutation‐based hypothesis testing and niche overlap analyses, we consistently found that diet composition and trophic niche structure were primarily shaped by ontogeny rather than sex. Juvenile ASTs exhibited a diverse, invertebrate‐dominated diet, whereas larger individuals progressively transitioned toward strong piscivory, resulting in increasing dietary specialization and trophic divergence among life stages. This pattern is consistent with size‐structured trophic differentiation widely documented in ectothermic vertebrates (Werner and Gilliam 1984). Clear ontogenetic changes in prey use were evident. Juveniles consumed a broad range of invertebrates, whereas fish became increasingly dominant in subadult and adult diets. Multivariate analyses of dietary composition supported this pattern, with juveniles occupying a distinct region and successive age classes forming a trajectory toward fish‐dominated diets. Together, these results indicate a shift from generalized feeding in early life stages to piscivory in larger individuals.

Ontogenetic changes in diet were further accompanied by a progressive reduction in dietary diversity and niche breadth. Dietary richness declined with increasing body mass, and both multivariate dispersion and pairwise Bray–Curtis dissimilarities revealed higher variability in juvenile diets and increasing dietary convergence among older age classes. Consistent with this pattern, niche overlap analyses showed low overlap between juveniles and adults but high overlap among subadult and adult stages, indicating that trophic niches become increasingly constrained and similar as ASTs grow. Such patterns are characteristic of size‐structured food webs and reflect ontogenetic increases in body size, swimming performance, prey handling capacity, and energetic demands, which collectively expand access to energetically profitable prey such as fish (Werner and Gilliam 1984; Woodward and Hildrew 2002).

### 4.2 Mechanisms Underlying Ontogenetic Dietary Differentiation

The absence of detectable dietary differences between male and female ASTs is consistent with the lack of pronounced sexual dimorphism observed during field surveys in the Jewellery Island region. In turtles, sex‐specific trophic differentiation is often associated with sexual morphological differences that lead to divergent foraging strategies and prey accessibility (Plummer and Farrar 1981; Aresco et al. 2009). The apparent morphological similarity between male and female ASTs therefore provides a plausible explanation for the absence of sex‐related trophic segregation observed in this study, reinforcing the conclusion that ontogeny, rather than sex, represents the primary axis structuring dietary variation in this species.

Ontogenetic dietary shifts in ASTs are likely closely linked to age‐specific habitat use and spatial ecology. Previous telemetry studies in the Ussuri River have shown that subadult and adult ASTs differ markedly in home range size and movement behavior, with subadults preferentially occupying shallow, slow‐flowing channels rich in aquatic vegetation, whereas adults exploit a broader range of habitats, including open and deeper sections of the main river channel (Hou and Shi 2024, 2025). Although juvenile habitat use could not be directly quantified due to telemetry limitations, it is reasonable to infer that juveniles exhibit spatial preferences similar to those of subadults. Such age‐related habitat partitioning likely contributes to the observed dietary differences by exposing different life stages to distinct prey assemblages.

Vegetated, low‐flow channels and marginal habitats along the river–land interface support high abundances of benthic and epibenthic invertebrates, including freshwater snails, aquatic insect larvae, and small crustaceans. These habitats therefore provide ample prey for juvenile and subadult ASTs, consistent with the high diversity and frequency of invertebrate prey observed in their diets. In contrast, adult ASTs, with greater swimming ability, bite force, and predator avoidance capacity (Hou and Shi 2024), are able to exploit a broader range of aquatic habitats rich in energetically profitable fish, potentially leading to a pronounced ontogenetic shift toward piscivory.

The sharp decline in consumption of freshwater snails (primarily *Viviparus chui*) with ontogeny reflects both prey traits and predator capabilities. Juvenile *V. chui* are small‐bodied with thin shells, making them easily handled and ingested by juvenile ASTs. In contrast, adult snails grow larger and develop thicker shells, reducing their accessibility for larger turtles. Observationally, adult ASTs in the Ussuri River do not exhibit the highly developed jaw musculature and broad heads seen in other freshwater turtles specialized in molluscivory (Ernst and Lovich 2009; Plummer 1977a, 1977b), which is consistent with their dietary shift toward more abundant and energetically profitable fish. Similar ontogenetic limitations on prey handling and diet composition have been documented in other freshwater turtles (Bouchard and Bjorndal 2006), supporting the idea that prey morphology interacts with predator ontogeny to shape diet composition. At the same time, larger ASTs gain access to abundant fish prey that offer substantially higher energetic returns. The combination of increasing handling constraints for snails and high availability of fish likely drives the rapid transition from mollusk‐dominated to fish‐dominated diets, consistent with predictions from optimal foraging theory (Schoener 1971; Werner and Hall 1974).

Patterns observed for other prey groups further support a size‐mediated mechanism. Aquatic decapods and aquatic insect larvae maintained moderate importance in juvenile and subadult diets but decreased markedly in adults, likely reflecting differences in prey size, habitat association, and capture efficiency across life stages. In contrast, terrestrial invertebrates showed similar frequencies of occurrence across all age classes, suggesting largely opportunistic consumption rather than age‐specific prey selection.

### 4.3 Comparisons With Other Softshell Turtles

Dietary information remains limited for most softshell turtle species, with detailed quantitative studies available for only a small number of taxa and systems (Moll and Moll 2004; Ernst and Lovich 2009). Existing studies on *Pelodiscus sinensis* and several Apalone species generally describe omnivorous or broadly carnivorous diets, in which aquatic invertebrates dominate and plant material is frequently consumed to varying degrees (Plummer and Farrar 1981; Mahoney and Lindeman 2016; Kong et al. 2022). In this context, the near‐exclusive reliance on animal prey across all life stages and the pronounced piscivory observed in adult ASTs distinguish the Ussuri River population from most previously studied softshell turtles.

While ontogenetic dietary shifts, such as increased fish consumption with growth, have been observed in some freshwater turtles (Bouchard and Bjorndal 2006), this transition is especially marked in ASTs. In the Ussuri River, fish became the dominant dietary component for ASTs at relatively moderate body sizes, and adults exhibited exceptionally high levels of piscivory, a pattern never documented in earlier softshell turtle studies. We suggest that this divergence reflects strong environmental context dependence in trophic ecology. The Ussuri River supports abundant, high‐quality prey resources—particularly diverse and numerically dominant fish assemblages (Bogutskaya et al. 2008)—which likely promote specialization on energetically profitable prey as turtles grow, consistent with predictions from optimal foraging theory (Schoener 1971; Werner and Hall 1974).

Together, the current and previous studies suggest that both ontogenetic stage and environmental context may influence trophic strategies in softshell turtles.

### 4.4 Implications for the Ussuri River Food Web

Ontogenetic trophic differentiation in ASTs likely has important consequences for the structure and functioning of the Ussuri River food web. Juvenile turtles, by consuming benthic invertebrates and freshwater snails, may influence invertebrate community composition and benthic energy pathways, whereas subadult and adult turtles function primarily as piscivorous predators. This stage‐specific partitioning of trophic roles implies that ASTs occupy multiple functional positions within the riverine food web over their lifetime, linking benthic and pelagic energy channels and potentially stabilizing prey populations through size‐structured predation.

The pronounced decline of ASTs populations in northeastern China therefore raises concerns about the loss of these functional roles. Because larger ASTs consume primarily fish, their removal could have uncertain consequences for fish community structure and competitive interactions. Similarly, the loss of juvenile ASTs may reduce predation pressure on benthic invertebrates, potentially affecting energy flow and nutrient dynamics (Polis 1984; Polis et al. 2000; Woodward and Hildrew 2002).

### 4.5 Conservation and Management Implications

Our findings highlight that effective conservation of ASTs depends on preserving the ecological integrity of the Ussuri River. The persistence of abundant fish and freshwater invertebrate communities—key trophic resources for different life stages of ASTs—relies on river habitats with high water quality and minimal human disturbance. Degradation of these conditions may rapidly undermine the trophic foundation necessary for ASTs' survival. In addition, strengthened legal protection is urgently needed to prohibit the capture and trade of this already critically imperiled species.

More broadly, the strong ontogenetic dietary shifts documented here suggest that ASTs should be regarded as functionally important predators within temperate river ecosystems rather than ecologically redundant components. Incorporating trophic ecology into conservation assessments may therefore improve predictions of ecosystem‐level consequences following population declines. Future studies integrating stable isotope analyses and long‐term monitoring of prey communities would further clarify the role of ASTs in mediating energy flow and community dynamics in large river systems such as the Ussuri River.

Although the present study provides robust evidence for ontogenetic dietary differentiation in ASTs, several limitations should nevertheless be acknowledged. Although 41 nonempty stomachs were sufficient to reveal clear ontogenetic dietary patterns, the sample size remained modest after subdivision into ontogenetic groups. This may have reduced statistical power to detect subtle effects, particularly weak sex‐related dietary differences or finer‐scale variation among adjacent size classes. In addition, dietary estimates for juvenile ASTs may be more sensitive to inter‐individual variation because this age class exhibited the broadest dietary niche. Despite these limitations, the strong consistency among descriptive dietary metrics, multivariate ordination, PERMANOVA, and niche overlap analyses indicates that the major patterns reported here are robust.

## 5 Conclusion

This study provides the first comprehensive evidence for pronounced ontogenetic dietary shifts in the Amur softshell turtle ( *Pelodiscus maackii* ) within a temperate large‐river system. Using complementary stomach content metrics, multivariate analyses, and niche overlap approaches, we demonstrate that trophic differentiation in this species is driven primarily by body size and developmental stage rather than sex. Juvenile turtles relied predominantly on benthic invertebrates, particularly freshwater snails, whereas subadult and adult individuals transitioned rapidly toward strong piscivory, accompanied by declining dietary richness and increasing trophic specialization.

These results highlight ontogeny as a central organizing axis of trophic ecology in ASTs and indicate that different life stages occupy distinct functional roles within the riverine food web. Such size‐structured trophic differentiation suggests that population declines or size‐selective losses may have disproportionate ecological consequences, even prior to complete extirpation. By linking individual development to food‐web structure and ecosystem functioning, our findings underscore the importance of preserving both population size and natural size structure in conservation strategies for this critically imperiled freshwater turtle.

## Author Contributions

**Xiaochen Hou:** conceptualization (lead), data curation (lead), formal analysis (lead), investigation (lead), methodology (lead), project administration (lead), resources (lead), software (lead), supervision (lead), validation (lead), visualization (lead), writing – original draft (lead), writing – review and editing (lead).

## Funding

The author has nothing to report.

## Ethics Statement

Institutional Review Board statement: Fieldwork was carried out in strict accordance with the guidelines of the Animal Research Ethics Committee of the Hainan Provincial Education Centre for Ecology and Environment, Hainan Normal University (HNECEE 2016‐005), which conforms to the laws of China. No turtles were euthanized or injured in this study.

## Conflicts of Interest

The author declares no conflicts of interest.

## Supporting information


**Figure S1:** Opportunistic photographs documenting *Pelodiscus maackii* observed in local markets during field surveys in the Ussuri River region.
**Figure S2:** Multivariate dispersion and pairwise dietary dissimilarity among ontogenetic stages. (a) Distances of individuals to group centroids in Bray–Curtis space (beta dispersion). (b) Pairwise Bray–Curtis dissimilarities among individuals from different ontogenetic stages.
**Table S1:** Detailed prey taxa identified in stomach contents of Amur softshell turtles and their frequencies of occurrence across ontogenetic stages.
**Table S2:** Pairwise PERMANOVA results comparing dietary composition among ontogenetic groups of *Pelodiscus maackii* based on Bray–Curtis dissimilarities.

## Data Availability

The data and R code supporting the findings of this study are available in the Dryad Digital Repository at http://doi.org/10.5061/dryad.z8w9ghxw3.
