# Supplementary material for: Ontogenetic Trophic Differentiation in the Amur Softshell Turtle (Pelodiscus maackii) From a Temperate Large‐River System
Source: Ecol Evol. 2026 Aug 26;16(9):e74221. doi: 10.1002/ece3.74221 (PMC13519293; doi:10.1002/ece3.74221)
Supplement: Supplementary file 1 [file ECE3-16-e74221-s001.docx]

**Figure S1.** Opportunistic photographs documenting Pelodiscus maackii observed in local markets during field surveys in the Ussuri River region.

| **TABLE S1** Detailed prey taxa identified in stomach contents of Amur softshell turtles and their frequencies of occurrence across ontogenetic stages. | | | | | |
| --- | --- | --- | --- | --- | --- |
| **Dietary Group** | **Taxon** | **FO (%) Juvenile** | **FO (%) Small subadult** | **FO (%) Large subadult** | **FO (%) Adult** |
| Fish | *Hemiculter Leucisclus* — species | 10 | 29 | 33 | 40 |
|  | *Squalidus wolterstorffi* — species | 20 | 29 | 44 | 47 |
|  | *Tachysurus fulvidraco* — species | 10 | 14 | 33 | 33 |
|  | *Pseudobagrus ussuriensis* — species | 0 | 0 | 11 | 20 |
|  | *Acheilognathus macropterus* — species | 20 | 43 | 44 | 40 |
|  | *Sarcocheilichthys nigripinnis* — species | 0 | 43 | 33 | 33 |
|  | *Erythroculter* spp. — genus | 0 | 0 | 11 | 20 |
|  | *Cyprinus carpio* — species | 0 | 0 | 0 | 13 |
|  | *Perccottus glenii* — species | 0 | 14 | 11 | 20 |
|  | *Rhynchocypris lagowskii* — species | 0 | 0 | 11 | 13 |
|  | *Lota lota* — species | 0 | 0 | 11 | 13 |
|  | *Megalobrama amblycephala* — species | 0 | 0 | 0 | 20 |
|  | *Carassius auratus* — species | 0 | 0 | 11 | 13 |
|  | Unidentified fish remains — unknown | 30 | 57 | 56 | 53 |
| Aquatic decapods | *Macrobrachium* spp.— genus | 70 | 57 | 22 | 13 |
|  | *Cambaroides* spp.— genus | 0 | 0 | 11 | 7 |
| Aquatic insect (larvae) | Ephemeroptera larvae — order | 60 | 29 | 11 | 0 |
|  | Odonata larvae — order | 20 | 43 | 56 | 27 |
|  | Unidentified insect larvae remains — unknown | 30 | 14 | 22 | 7 |
| Freshwater snails | *Cipangopaludina ussuriensis* — species | 90 | 71 | 33 | 7 |
|  | *Semisulcospira* spp.— genus | 10 | 0 | 11 | 0 |
| Terrestrial invertebrates | Orthoptera spp.— order | 0 | 14 | 22 | 13 |
|  | Lepidoptera spp.(larvae or adult)— order | 10 | 14 | 22 | 13 |
|  | Coleoptera spp.— order | 20 | 0 | 11 | 0 |
|  | Odonata spp.— order | 10 | 29 | 33 | 20 |
|  | Ephemeroptera spp.— order | 30 | 14 | 0 | 7 |
|  | Haplotaxida spp.— order | 10 | 0 | 11 | 7 |
|  | Unidentified invertebrates remains — unknown | 30 | 29 | 22 | 20 |

| **TABLE S2** Pairwise PERMANOVA results comparing dietary composition among ontogenetic groups of Pelodiscus maackii based on Bray–Curtis dissimilarities. | | | | |
| --- | --- | --- | --- | --- |
| Comparison | F value | R² | P-value | Adjusted P-value(Holm) |
| Juvenile vs Small subadult | 8.349 | 0.358 | 0.001 | 0.006 |
| Juvenile vs Large subadult | 15.662 | 0.480 | 0.001 | 0.006 |
| Juvenile vs Adult | 66.312 | 0.742 | 0.001 | 0.006 |
| Small subadult vs Adult | 17.103 | 0.461 | 0.001 | 0.006 |
| Large subadult vs Adult | 4.028 | 0.155 | 0.011 | 0.022 |
| Small subadult vs Large subadult | 1.642 | 0.105 | 0.153 | 0.153 |
| Notes: F and R² values were obtained from pairwise PERMANOVA analyses based on Bray–Curtis dissimilarities. R² represents the proportion of explained variation. P-values were adjusted for multiple comparisons using the Holm correction. | | | | |

### ****FIGURE S2**** Multivariate dispersion and pairwise dietary dissimilarity among ontogenetic stages.(a) Distances of individuals to group centroids in Bray–Curtis space (beta dispersion).(b) Pairwise Bray–Curtis dissimilarities among individuals from different ontogenetic stages.
